# Treatment of bony Bankart lesion in geriatric patient with reverse total shoulder arthroplasty using a half-wedge augmented glenoid component: a case report

**DOI:** 10.1016/j.xrrt.2023.04.003

**Published:** 2023-04-25

**Authors:** Albert T. Anastasio, Mikhail Bethell, Chinedu Okafor, Jay Levin, Oke Anakwenze

**Affiliations:** a Department of Orthopedic surgery, Duke University Hospital, Durham, NC, USA; b Duke University School of Medicine, Durham NC, USA

**Keywords:** Bony Bankart, Reverse total shoulder arthroplasty, Glenoid augment, Shoulder surgery, Half-wedge, Bone loss

Bony Bankart and Hill-Sachs lesions are both common sequela to traumatic, anterior glenohumeral joint dislocation with incidence reports ranging from 5.4%-44% for bony Bankart injuries and 31%-93% for Hill-Sachs lesions.^3^^,^^5^^,^^8^^,^^10^^,^^11^^,^^15^^,^^18^^,^^21^, ^22^, ^23^^,^^25^^,^^28^^,^^31^^,^^32^ Anterior shoulder instability associated with bony Bankart lesions poses a unique clinical challenge that has historically been managed with open reduction and internal fixation of the fracture or bone augmentation or transfer at the anteroinferior glenoid.^19^^,^^26^ When occurring in the geriatric population, reverse shoulder arthroplasty (RSA) can be a viable treatment option. However, in the setting of significant bony Bankart and glenoid bone loss, RSA performed without addressing the bony defect may render the baseplate at an increased risk of failure due to unstable fixation. Moreover, treatment with open reduction and internal fixation for these patients poses additional concerns, given osteoporosis and poor bone quality as well as the high prevalence of pre-existing rotator cuff insufficiency.^2^ Finally, bone graft augmentation to the anterior glenoid presents technical challenges with regards to adequate screw placement, avoidance of glenoid baseplate screws, and achieving successful incorporation of bone graft augment, especially in geriatric patients.

To address these challenges with existing treatment modalities, we present a novel case of a geriatric patient with anterior shoulder instability resulting from an acute, traumatic anterior dislocation with a large Hill-Sachs fracture and concomitant bony Bankart fracture treated with RSA and a one-half glenoid wedge (half-wedge) augment placed in the anterior inferior defect.

## Case report

### Preoperative course

**Figure 1:**
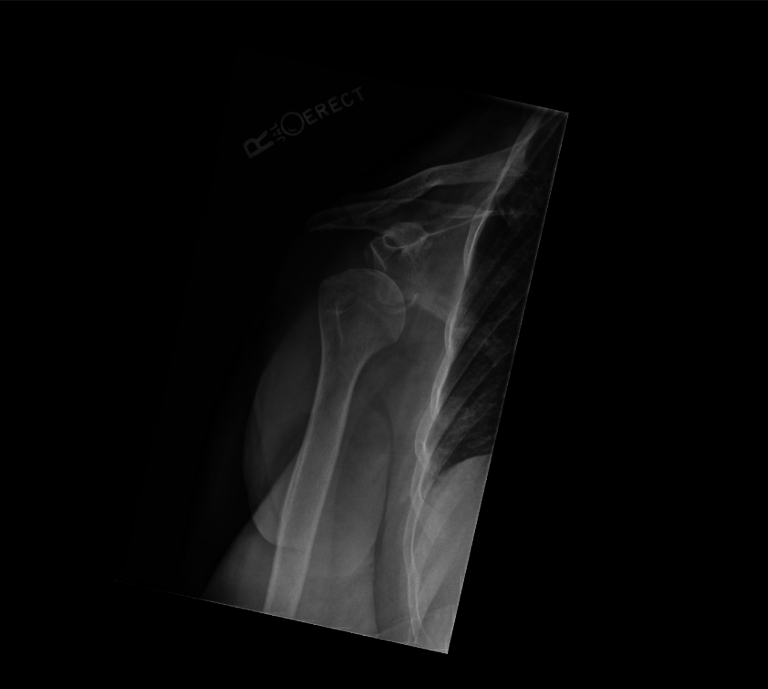
Preoperative upright anteroposterior radiograph of the right shoulder illustrating persistent anteroinferior dislocation of the Right glenohumeral joint. A curvilinear density along the inferior aspect of the glenoid was consistent with bony Bankart fracture.

An 83-year-old right-hand–dominant female presented to the emergency department after a fall at home with symptoms of severe right shoulder pain, swelling, and an inability to elevate her right arm. Shoulder radiographs demonstrated an anterior-inferior glenohumeral dislocation with a bony Bankart lesion (Fig. 1). Closed reduction was successful, and she was placed in a sling. Despite this, on follow-up, her shoulder was shown to be subluxated on radiographs and advanced imaging was ordered.

**Figure 2:**
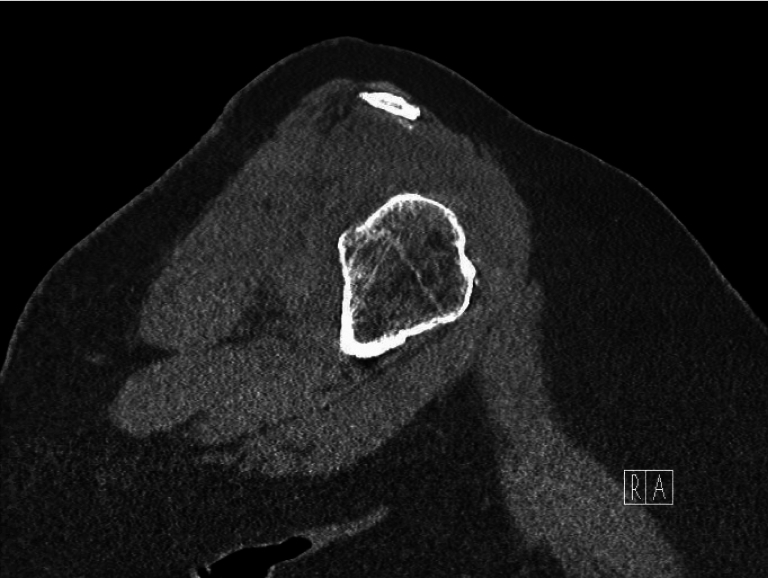
Preoperative CT cut of the right shoulder illustrating a subtle, minimally depressed fracture of the posterosuperior aspect of the humeral head and acute comminuted fractures of the anteroinferior glenoid within the joint space and inferior subluxation of the humeral head with respect to the glenoid was noted intraoperatively, although not visible on the CT scan. *CT*, computed tomography.

**Figure 3:**
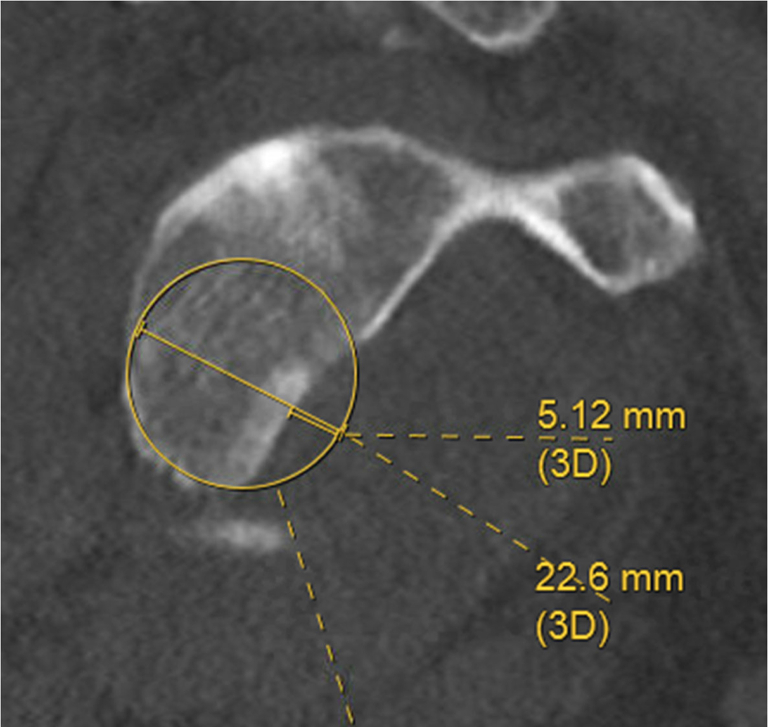
Illustration of the perfect circle technique, carried out on the preoperative CT sagittal oblique view of the right shoulder. Perfect circle technique revealed a glenoid bone loss of 22.7%. This image also reveals a fracture fragment anteroinferior to the glenoid. *CT*, computed tomography.

Computed tomography (CT) showed sequela of anterior-inferior right shoulder dislocation with mildly depressed Hill-Sachs lesion to the humerus and comminuted displaced bony Bankart fracture. These injuries included comminuted fractures of the anteroinferior glenoid with multiple fracture fragments present within the joint space, with fragments within the axillary pouch and at least 2 pieces superomedial to the humeral head, the largest of which measured 1.5 cm in the maximal dimension (Fig. 2). We used the best-fit perfect circle technique on the CT sagittal oblique view to calculate the glenoid bone loss (Fig. 3).

Based on this CT scan, a glenoid bone loss of 22.7% was noted as well as an off-track Hill-Sachs humeral head lesion. There was also confirmation of inferior subluxation of the humeral head with respect to the glenoid as well as moderate glenohumeral arthrosis.

After consideration of a variety of factors, the decision was made to proceed with RSA with glenoid augmentation. Other surgical options that were discussed included Latarjet procedure, distal tibia allograft, or iliac crest bone graft for glenoid augmentation in addition to hemiarthroplasty or humeral head allograft for the Hill-Sachs defect. Unfortunately, all of these procedures require a healthy rotator cuff, excellent bone quality, and a more involved physical therapy regimen postoperatively. Given the degree of shoulder dislocation in a geriatric patient, we assumed an incompetent rotator cuff, and thus RSA was thought to have the lowest chance of reoperation or subsequent instability event.

### Preoperative planning

**Figure 4:**
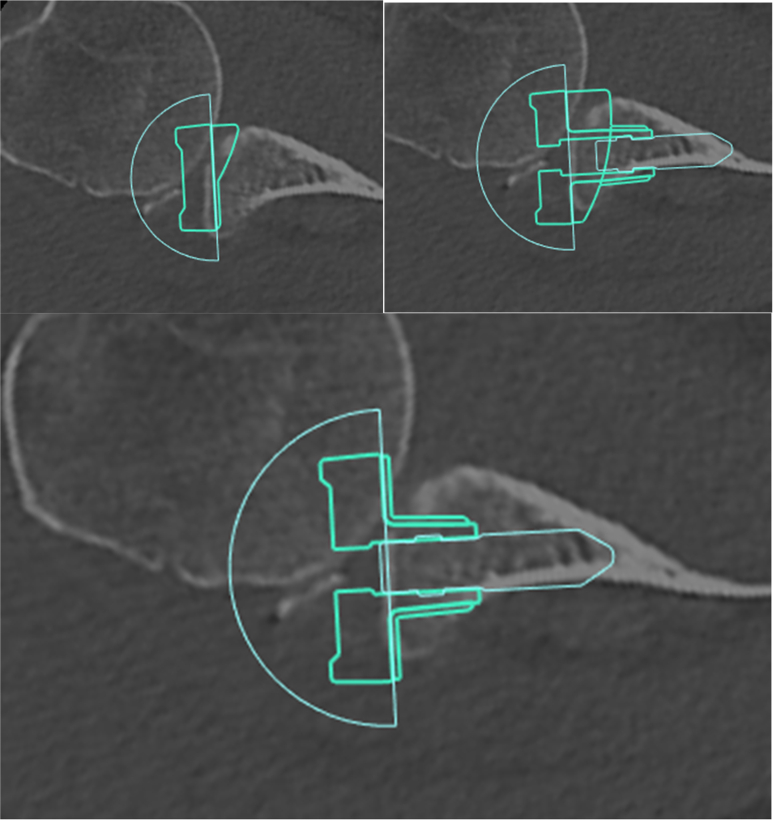
Preoperative templating for RSA, using Stryker Blueprint 3D planning software (Kalamazoo, MI, USA). We elected to use a half-wedge glenoid augment (Stryker, Kalamazoo, MI, USA) to address the glenoid deficiency, which allowed for filling in the bony glenoid defect without resecting as much bone as would be required for a full backside wedge to fully seat the implant. *RSA*, reverse shoulder arthroplasty.

With regards to preoperative planning, we used Stryker Blueprint 3-Dimensional (3D) planning software (Kalamazoo, MI, USA) based on the CT imaging. The 3D reconstruction confirmed anterior glenoid bone loss. We then templated for RSA, determining the appropriate size of the humerus and glenoid implants (Fig. 4). Based on preoperative planning, we elected to use a half-wedge glenoid augment (Stryker, Kalamazoo MI, USA) to address the glenoid deficiency. Use of a half wedge allowed for filling in the bony glenoid defect without resecting as much bone as would be required for a full backside wedge to fully seat the implant. Moreover, we felt that a standard glenoid baseplate would have left a large amount of the glenoid uncovered, unless the implant was significantly medialized, thus resulting in substantial bony resection.

### Operative course

The patient underwent RSA through a standard deltopectoral approach. The bicipital groove was unroofed, revealing a tendinopathic biceps tendon, which was ultimately addressed with biceps tenodesis. The posterior and superior rotator cuff was then visualized and noted to be torn. We proceeded to release the subscapularis tendon, anterior and inferior capsular structures, and the posterior band of the inferior glenohumeral ligament. A large Hill-Sachs defect of 21.7 mm was noted. The humeral head was then cut at the appropriate inclination to approximate the patient’s natural retroversion.

**Figure 5:**
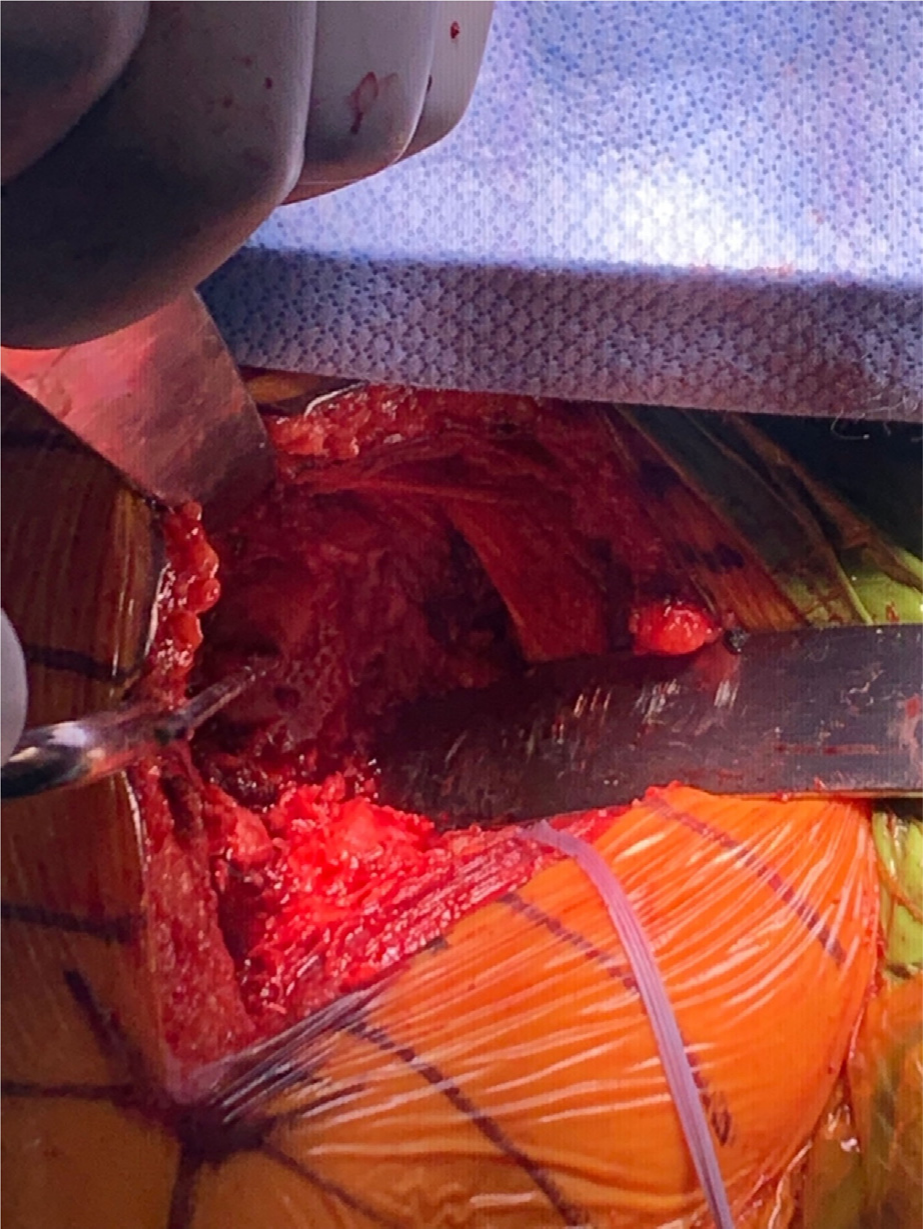
Visualization of the glenoid defect seen after exposure through the deltopectoral approach and capsular/tendinous release.

**Figure 6:**
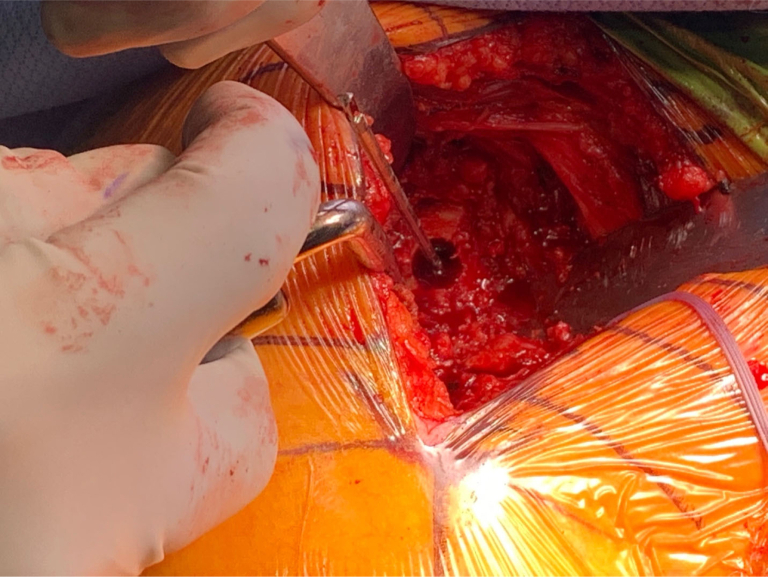
Glenoid preparation was carried out with central hole drilled prior to placement of the metal augment.

**Figure 7:**
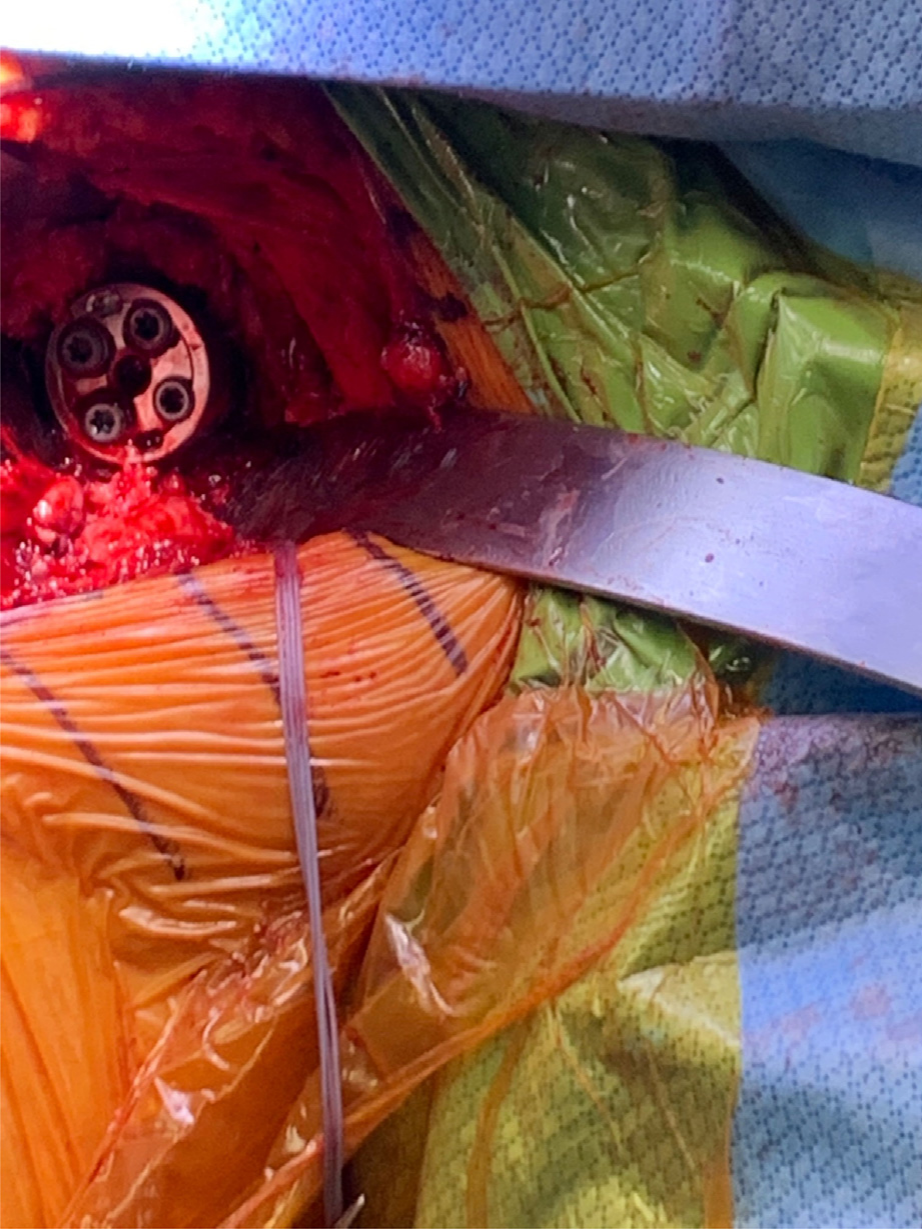
The augmented baseplate was then implanted with anteroinferior wedge to address bony Bankart.

**Figure 8:**
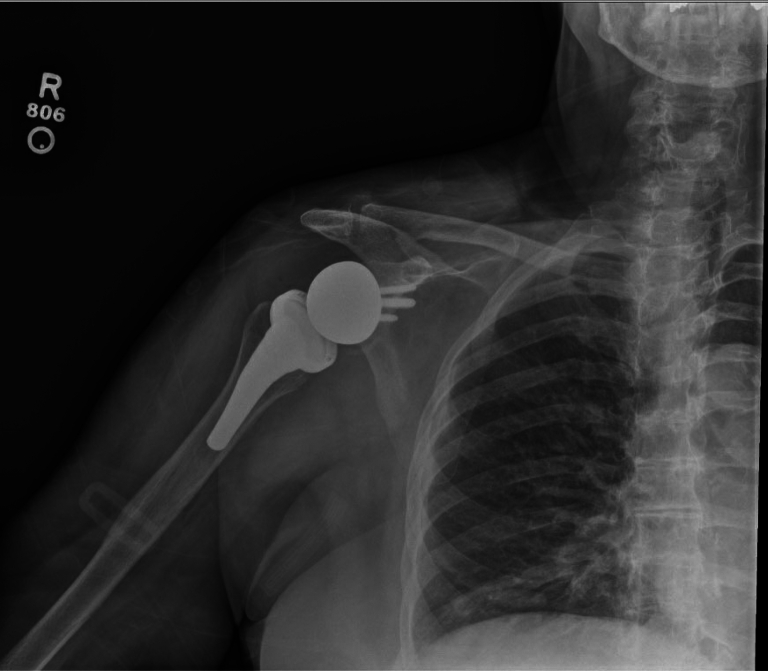
Immediate postoperative radiograph depicting interval postsurgical changes of right RSA. The hardware appeared intact without peripheral lucency. *RSA*, reverse shoulder arthroplasty.

Attention was then shifted to the glenoid, which revealed a large anterior glenoid defect, consistent with preoperative imaging. The glenoid defect was exposed carefully (Fig. 5). The glenoid and the defect were prepped adequately for the insertion of a half-wedge augmented baseplate (Fig. 6). The augment was placed anteroinferiorly after preparing the glenoid using the manufacturing instrumentation (Fig. 7). A glenosphere was inserted into the baseplate and the humeral component was introduced following a standard procedure. Radiographs demonstrated satisfactory RSA with metal augmentation of the glenoid defect (Fig. 8).

### Postoperative course

**Figure 9:**
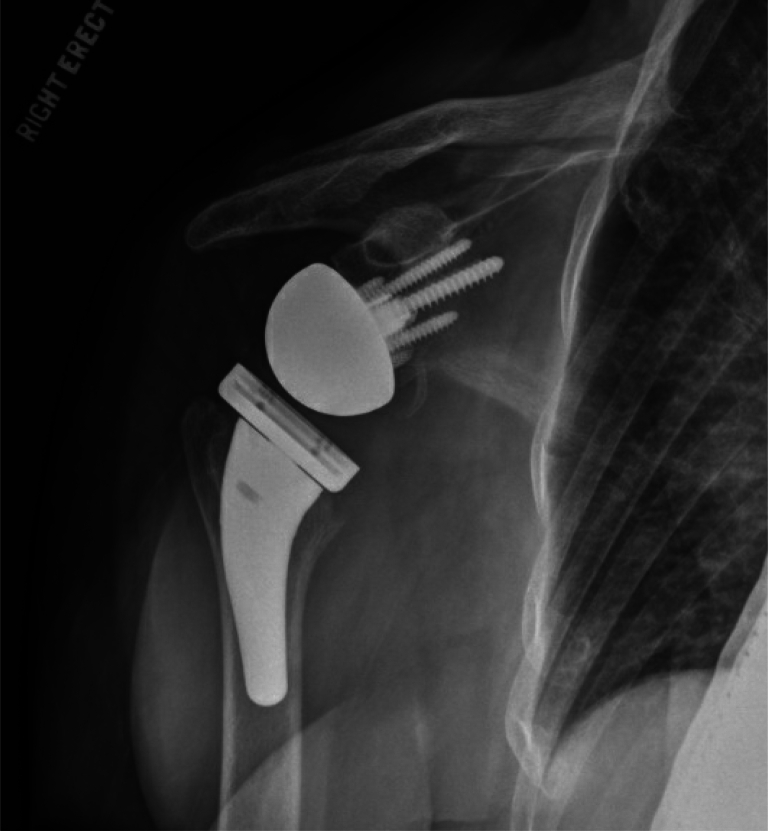
Upright radiograph of the right shoulder at the 2-week follow-up illustrating redemonstration of right reverse shoulder arthroplasty with no radiographic evidence of peri-hardware lucency or malalignment. The prosthesis appeared well-seated and no periprosthetic fracture was noted.

**Figure 10:**
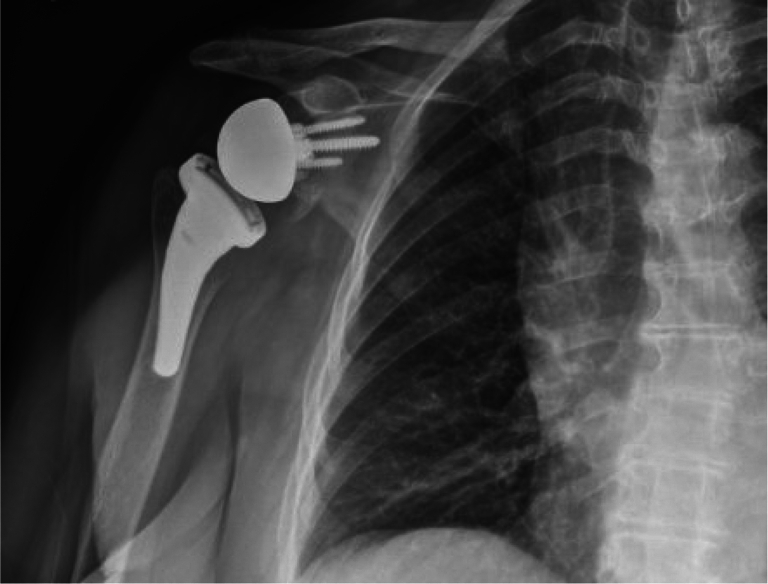
Upright radiograph of the right shoulder at the 2-year follow-up illustrating RSA without evidence of hard failure or periprosthetic lucency, bony fracture, and preserved joint spaces. *RSA*, reverse shoulder arthroplasty.

Immediate postoperative imaging indicated intact hardware without evidence of complication. Postoperative radiographs at 2 weeks (Fig. 9) and 2 years (Fig. 10) postsurgery demonstrated similar findings. Postoperative protocols included standard physical therapy with 6 weeks of nonweight bearing in a sling with passive range of motion. This was followed by an active assisted range of motion protocol from 6-12 weeks. At the 12-week mark, an active range of motion protocol was instituted, followed by therapist-lead engagement in light strengthening activities. A lifetime 10-pound overhead weight limit was discussed with the patient.

Two-year status postsurgery, the patient overall reports doing well, with physical examination indicating forward flexion to 170 degrees, internal rotation to the beltline, negative drop arm sign, and external rotation strength of 4/5. Patient-Reported Outcomes Measurement Information System physical function and pain interference scores revealed improvement from the preoperative to the postoperative period.

## Discussion

Joint concavity compression from the dynamic stabilizers at the shoulder in addition to support from the bony structures at the articulation between the humeral head and glenoid provide a large portion of the stability of the shoulder joint. Failure of the bony structures often accompanies anteroinferior shoulder dislocations, resulting in Hill-Sachs and bony Bankart lesions. These lesions can arise independently but may also occur concomitantly. Horst et al^9^ demonstrate a high co-occurrence rate of these 2 injuries, concluding that if a patient suffers either lesion, they are 11 times more likely to also present with the accompanying pathology. The presence of these co-occurring pathologies caused a markedly increased risk for subsequent glenohumeral instability.

With regard to management, when Hill-Sachs lesions and bony Bankart fractures are treated nonoperatively, high rates of recurrent anterior shoulder instability have been reported.^29^ In younger patients with Hill-Sachs and bony Bankart injuries and without glenohumeral arthrosis, soft tissue and bony repair options such as remplissage and Latarjet exist and can be successful in mitigating the risk of subsequent dislocation.^1^^,^^4^^,^^6^^,^^16^^,^^20^ Unfortunately, surgical options are limited in the setting of dislocation in geriatric patients where arthritic change is predictably present.^17^ Thus, we present a novel solution for glenoid bone loss and shoulder instability with concomitant glenohumeral osteoarthritis in a geriatric patient through RSA with an augmented glenoid base plate.

Indications for RSA with augmented glenoid base plate vs. other forms of surgical treatment in geriatric patients with shoulder instability, with associated unipolar or bipolar bone loss, are unclear and poorly investigated in the literature. Multiple factors when considering RSA in this setting are paramount to optimal success of the chosen treatment option, including patient health and activity, ability to engage and comply with respective therapy protocols, presence or absence of arthritis, and the status of the rotator cuff.

Literature on geriatric patients with shoulder instability is scant. Domos et al presented a cohort of 99 patients aged more than 40 years receiving Latarjet at a mean follow-up period of 13 years (range, 3-23 years). At the time of final follow-up, 94% of the patients did not exhibit recurrence of instability, 90% were satisfied or very satisfied with their outcomes, and 54% returned to their preinjury level of activity. However, 9% of patients required reoperation.^7^ In addition, while all patients were aged more than 40 years, the mean age was 46 years. Thus, this population represents a very young cohort in comparison to the geriatric or octogenarian population with poor bone quality or ability to engage in extensive postoperative rehabilitation.

Thus, in the setting of anterior instability, glenoid bony defect, and pre-existing arthritis in the geriatric patient, operative management may increasingly involve RSA.^24^^,^^30^ A robust literature already exists for the use of RSA in the setting of proximal humerus fracture and for chronic shoulder dislocations.^27^ RSA for anterior shoulder instability has demonstrated improved range of motion, pain, and patient-reported satisfaction.^12^^,^^14^^,^^27^

Baseplate wedges have traditionally been employed to manage patients with glenoid deficiency in the setting of rotator cuff arthropathy or glenohumeral arthritis. Wedges may be used to prevent excessive bone removal, to restore the joint line, or to better tension the posterosuperior rotator cuff. Outcomes for these indications have been satisfactory as noted by Levin et al^13^ who noted that there were greater postoperative improvements in active range of motion and clinical outcomes in the augment baseplate cohort in comparison to the standard nonaugment baseplates. However, in the present scenario, where Hill-Sachs and bony Bankart lesions coexist with acute anteroinferior shoulder dislocation, treatment options have not been fully investigated. We recommend careful preoperative planning with CT-based templating to determine glenoid and humerus implant fit and confirms the size of the baseplate needed. Additionally, preoperative templating allows for careful consideration of glenoid morphology and for determination of the amount and angle of any areas of bony impingement. Moreover, the required bony resections can be planned, the degree of humeral lengthening and lateralization can be templated, and the decision can be made if wedge augmentation may be needed to fill bony defects.

With regards to technical considerations for these potentially challenging cases, we recommend use of a large incision to decrease tissue tension, soft tissue paralysis, and a cautious yet adequate deltoid release to allow the humerus to fall posterior to the glenoid to more optimally visualize the osseus anatomy. Furthermore, we use a relatively substantial inferior capsular release off the humerus and the glenoid as well as a proper release and development of the subscapularis recess to allow for adequate exposure of the anterior defect after retractor placement. An appropriately large head cut and adequate osteophyte resection will further facilitate implant placement. In our hands, the half wedge is typically preferred due to less bone reaming and the ability to fit within the glenoid fracture deficiency which usually is too acute of an angle for a traditional wedge augment.

## Conclusion

The present case provides an insight into an uncommon use of RSA-wedged baseplate to treat concomitant Hill-Sachs lesions, bony Bankart fractures, and resultant shoulder instability in the setting of acute anteroinferior shoulder dislocation in a geriatric patient. This case gives further consideration to the ever-expanding indications for RSA. Further investigation is warranted to continue to optimize treatment options for the challenging clinical scenario presented here, where Hill-Sachs and bony Bankart lesions co-occur in a geriatric patient in the setting of an acute anteroinferior shoulder dislocation.

## Disclaimers:

Funding: No funding was disclosed by the authors.

Conflicts of interest: The authors, their immediate families, and any research foundation with which they are affiliated have not received any financial payments or other benefits from any commercial entity related to the subject of this article.

Patient consent: The patient was informed that data and imaging concerning their case would be submitted for publication and the patient consented. All identifying information has been removed.
